# Effect of low-dose tadalafil once daily on glycemic control in patients with type 2 diabetes and erectile dysfunction: a randomized, double-blind, placebo-controlled pilot study

**DOI:** 10.1186/s13098-022-00825-w

**Published:** 2022-04-21

**Authors:** Min-Kyung Lee, Jae-Hyuk Lee, Seo-Young Sohn, Seo Yeon Lee, Tae-Yoong Jeong, Sae Chul Kim

**Affiliations:** 1 Division of Endocrinology and Metabolism, Department of Internal Medicine, Myongji Hospital, Hanyang University College of Medicine, Goyang-si, Republic of Korea; 2 Department of Urology, Myongji Hospital, Hanyang University College of Medicine, Goyang-si, Republic of Korea

**Keywords:** Tadalafil, Glycemic control, Erectile dysfunction, Type 2 diabetes

## Abstract

**Background:**

Phosphodiesterase type 5 inhibitors restore nitric oxide signaling, that plays a significant role in erectile function, and appears to counteract insulin resistance in animal and human models. This study was aimed to evaluate the glycemic and metabolic effects of low-dose tadalafil once daily in patients with type 2 diabetes and erectile dysfunction.

**Methods:**

A 6-month, randomized, double-blind, placebo-controlled pilot trial was conducted. Eligible patients were randomly assigned in a ratio of 2:1 to the tadalafil 5 mg and placebo groups; all patients received either tadalafil or placebo once a day. The primary efficacy endpoint was the absolute change in glycated hemoglobin (HbA1c) levels during the 6-month study period. The secondary efficacy endpoints included metabolic parameters and erectile function.

**Results:**

Of the 68 patients who completed this study, 45 and 23 patients were allocated to the tadalafil and placebo groups, respectively. The mean HbA1c level was significantly different between the groups over the 6-month study period (*P* = 0.021). After 6 months of treatment, the HbA1c decrement in the tadalafil group was greater than that in the placebo group (− 0.14 ± 0.53% vs. 0.20 ± 0.69%, *P* = 0.030). The International Index of Erectile Function-5 scores improvement was significantly greater in the tadalafil group than in the placebo group at 6 months (*P* = 0.003).

**Conclusion:**

This prospective pilot study showed that low-dose tadalafil administered once a day was effective in improving glycemic control and erectile function in patients with type 2 diabetes and erectile dysfunction.

*Trial registration* KCT0005666

## Background

Erectile dysfunction (ED) is a common complication of diabetes that is underdiagnosed and mostly left untreated [1]. Based on the International Index of Erectile Function (IIEF) score, the prevalence of ED is estimated to be > 50% in men with diabetes and approximately 3.5 times higher in men with diabetes than in those without [2]. Diabetic ED involves vascular and neurological mechanisms [3]. Nitric oxide (NO) plays a significant role in normal penile erection, and a lack of NO in diabetes triggers ED [4]. Phosphodiesterase type 5 (PDE-5) inhibitors selectively block the hydrolysis of cyclic guanosine monophosphate (cGMP) in the penile corpus cavernosum, thus enhancing NO-mediated smooth muscle relaxation, increasing blood flow to the penis, and facilitating erection [5]. PDE-5 inhibitors result in increased levels of cGMP and NO [6]. Exploratory studies have reported beneficial effects of PDE-5 inhibitors on ED in patients with type 2 diabetes (T2D) [7, 8].

Diabetes mellitus is a metabolic disorder characterized by chronic hyperglycemia resulting from defects in insulin secretion, insulin sensitivity, or both [9]. Insulin resistance (IR) is associated with the development of endothelial dysfunction and a reduction in NO bioavailability [10]. In humans, endothelial dysfunction is linked with diabetes mellitus via a mechanism that interrupts intracellular signaling pathways of insulin and NO production [11]. Endothelial NO mediates the insulin-induced effects by increasing intracellular levels of cGMP in human vascular smooth muscle cells [12]. The increase in glucose transport is stimulated by insulin via the endothelium-derived NO/cGMP pathway [13]. Reduced NO synthesis in endothelial cells contributes to impaired insulin action in patients with T2D [14]. Therefore, treatments that amplify NO/cGMP signaling could improve microvascular recruitment and muscle glucose uptake [15]. Moreover, recent evidence from animal models and in men with ED suggests that PDE-5 inhibition may have metabolic benefits [16]. Nevertheless, a meta-analysis of PDE-5 inhibitors studies showed little benefit in improving glycemic control [17].

Tadalafil, a drug used to treat ED, selectively inhibits PDE-5 in the penile corpus cavernosum; and is an important regulator of smooth muscle relaxation by elevating intracellular NO/cGMP levels [18]. Treatment with tadalafil 5 mg once a day (OAD) is highly effective, safe, and well-tolerated in patients with ED associated with T2D [19]. A recent meta-analysis confirmed that the chronic use of PDE-5 inhibitors is effective in improving endothelial function, measured using brachial artery flow-mediated dilation and endothelial markers in patients with T2D [20]. Furthermore, in a pilot study, tadalafil OAD increased basal insulin secretion in men with ED [21]. In another study, tadalafil improved peripheral microcirculation and glucose uptake in patients with T2D [22]. Overall, these preliminary studies suggest a therapeutic effect of chronic daily use of tadalafil on glycemic control.

We conducted a randomized clinical trial to evaluate the effect of the chronic use of low-dose tadalafil OAD on glycemic control, assessed its effect according to glycated hemoglobin (HbA1c) levels and other metabolic effects in patients with T2D and ED. Additionally, we assessed the long-term efficacy and safety of tadalafil in treating erectile function.

## Methods

### Study design

A randomized, double-blind, placebo-controlled pilot study was conducted to examine the glycemic and metabolic effects of 6-month treatment with tadalafil 5 mg OAD in patients with T2D and ED. Statistical criteria for sample size are not required in the pilot study. All patients were randomized to receive tadalafil or placebo and were instructed to use it OAD for 6 months at the same time every day. The allocation list was produced using dedicated software (ID-net™) via permuted-block randomization with 2:1 allocation and randomly sized blocks. The allocation details were blinded until statistical analysis was completed. All the patients were monitored during the entire study period. Treatment compliance was defined as the administration of at least 70% of the required dose between visits.

The study protocol was reviewed and approved by the institutional review board of Myongji Hospital (Approval no. MJH-16-038). Informed consent was obtained from all the participants when they were enrolled. The study was conducted following the protocol, ethical principles stated in the Declaration of Helsinki (revised in 2000), and applicable laws. This randomized clinical trial was registered at the Clinical Research Information Service (CRIS, http://cris.nih.go.kr), number KCT0005666.

### Study participants

Eligible men were recruited from the outpatient clinic of Myongji Hospital, Goyang-si, Gyeonggi-do, the Republic of Korea between January 2017 and November 2018. Men aged 35–75 years who had a regular sexual partner and had intercourse ≥ 1 time in the last month, patients with T2D and ED for > 1 year, patients with HbA1c level < 9%, and patients with no history of PDE-5 inhibitor use in the last 3 months were included in this study. ED was defined as a history of persistent inability to achieve or maintain an erection sufficient for satisfactory sexual performance. The exclusion criteria were as follows: use of exogenous insulin, thiazolidinediones, or nitrate; history of malignancy, high-risk cardiovascular disease, and chronic liver or kidney failure; and contraindications to tadalafil. In addition, concomitant medications (such as oral anti-hyperglycemic medications, anti-hypertensive drugs, and statins) were not permitted to change between 3 months before study initiation and 1 month after study completion.

### Measurements

The selection criteria were evaluated at baseline and a complete clinical record was obtained from each subject, including demographic characteristics, medical history, alcohol consumption, smoking status, and other medical conditions. Body mass index (BMI) was calculated as weight in kilograms divided by the square of height in meters. A single examiner measured waist circumference (WC) in the standing position, and blood pressure (BP) was measured twice using a standardized sphygmomanometer with a 5-min rest period.

Venous blood samples were collected in the morning after an overnight fast of > 8-h. The concentrations of fasting plasma glucose (FPG), insulin, C-peptide, HbA1c, total cholesterol (TC), high-density lipoprotein cholesterol (HDL-C), low-density lipoprotein cholesterol (LDL-C), triglycerides (TG), blood urea nitrogen (BUN), creatinine, aspartate transaminase (AST), and alanine transaminase (ALT) were measured. Homeostatic model assessment (HOMA) is a method for assessing β-cell function and IR from basal FPG and insulin or C-peptide concentrations [23]. The equations were simplified as HOMA-IR = (fasting plasma insulin × FPG)/22.5 [24]. The estimated glomerular filtration rate (eGFR) was calculated using the Modification of Diet in Renal Disease Study equation. The hexokinase method, enzymatic method, and homogenous enzymatic colorimetric test were used to measure FPG, TC, TG, HDL-C and LDL-C levels, respectively. HbA1c levels were measured using turbidimetric inhibition immunoassay. Biochemical variables were measured using a Cobas Modular 6000 analyzer series (Roche Diagnostics, Basel, Switzerland). These variables were checked at baseline and 6 months. Weight, WC, BP, FPG, insulin, C-peptide, and HbA1c levels were measured at 3 months.

### Questionnaires

The secondary measures of efficacy of erectile and voiding function included the IIEF-5 score and the International Prostate Symptom Score (IPSS). The IIEF-5, the abridged five-item version of the IIEF, was used to evaluate erectile function [25]. The possible IIEF-5 scores ranged from 5 to 25. The IPSS is a validated seven‐item questionnaire used to assess lower urinary tract symptoms (LUTS). All participants completed self-administered questionnaires at baseline and 3 and 6 months of treatment.

### Safety assessment

Safety and tolerability were evaluated through adverse event (AE) monitoring throughout the study. The study investigators obtained and recorded all observed or self-reported AEs and their severities (mild, moderate, or severe). The relationships between AEs and study medication were established prior to breaking the blind. All patients underwent laboratory tests, vital sign examinations, physical examinations, and 12-lead electrocardiography at the baseline and end of the trial. Drug adherence was measured using the medication possession ratio, defined as the total number of days covered by filled prescriptions divided by the total number of observation days.

### Statistical analysis

All analyses were prespecified. The study outcomes of both groups were compared using the Student’s *t*-test for continuous variables and Pearson’s Chi-square test for categorical variables. Data are expressed as mean ± SD or number (proportion). Repeated measures analysis of variance (ANOVA) was used to monitor the differences in HbA1c levels between the groups over the 6 months. Exploratory data analysis was used to investigate the absolute changes in HbA1c levels from baseline at 3 and 6 months in both groups*.* Absolute change in HbA1c was defined as the difference between baseline HbA1c levels and HbA1c levels at 3 and 6 months. Pearson’s correlation analysis was used to explore the correlations between absolute changes in HbA1c levels and other variables. Statistical significance was set at *P* < 0.05. All statistical analyses were performed using IBM SPSS (version 18.0; IBM, Armonk, NY, USA).

## Results

### Study execution and baseline characteristics of participants

**Fig. 1:**
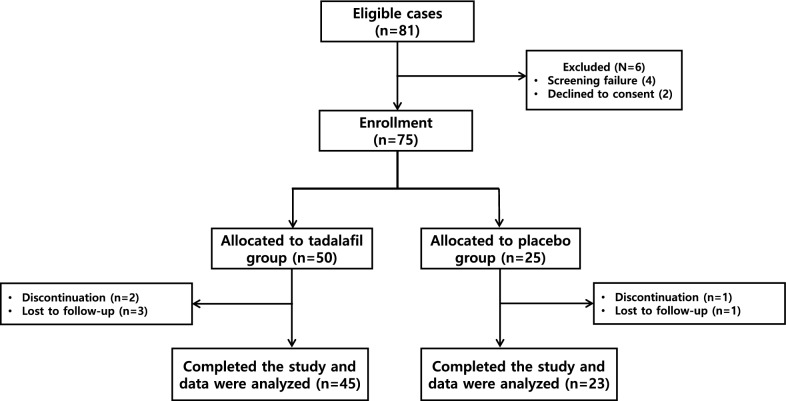
Consort flow diagram. The randomization process, treatment, and follow-up of study participants

81 patients with T2D and ED were randomized to receive tadalafil (*n* = 50) or placebo (*n* = 25) for 6 months. Subsequently, 5 and 2 patients from tadalafil and placebo groups, respectively, discontinued treatment. In the tadalafil group, 3 patients were lost to follow-up, and two discontinued treatment. In the placebo group, one patient was lost to follow-up, and one patient was discontinued. Overall, 45 and 23 subjects in the tadalafil and placebo groups completed the trial (Fig. 1). Baseline characteristics were well-matched between the groups at randomization and study completion (Table 1).

**Table 1:** Baseline characteristics and demographics

	Tadalafil (n = 45)	Placebo (n = 23)	*P-*value^*^
Age (years)	61.80 ± 7.25	58.87 ± 8.99	0.151
IIEF-5	10.47 ± 4.55	9.57 ± 4.11	0.428
IPSS	13.98 ± 5.77	14.47 ± 8.08	0.231
Waist circumference (cm)	86.89 ± 8.96	89.67 ± 6.21	0.187
Body mass index (kg/m^2^)	25.51 ± 3.05	26.59 ± 3.69	0.202
Systolic BP (mmHg)	127.84 ± 14.51	129.70 ± 17.57	0.645
Diastolic BP (mmHg)	87.34 ± 11.74	86.68 ± 8.73	0.538
HbA1c (%)	6.83 ± 0.77	6.77 ± 0.58	0.747
Fasting plasma glucose (mg/dL)	128.02 ± 24.95	120.70 ± 18.34	0.203
Insulin (IU/mL)	9.13 ± 9.14	10.04 ± 8.66	0.694
C-peptide (ng/mL)	1.77 ± 0.92	1.70 ± 0.77	0.686
HOMA-IR	3.05 ± 3.32	2.92 ± 2.51	0.870
Total cholesterol (mg/dL)	142.31 ± 28.05	140.43 ± 31.98	0.804
Triglyceride (mg/dL)	140.40 ± 123.55	144.61 ± 54.57	0.877
HDL-C (mg/dL)	44.42 ± 8.13	43.87 ± 9.50	0.803
LDL-C (mg/dL)	76.89 ± 23.32	74.48 ± 26.29	0.701
BUN (mg/dL)	16.10 ± 4.14	15.36 ± 5.84	0.549
Creatinine (mg/dL)	1.0 ± 0.207	1.01 ± 0.29	0.858
eGFR (mL/min/1.73 m^2^)	80.88 ± 14.96	83.49 ± 22.39	0.618
AST (IU/L)	27.09 ± 10.38	27.91 ± 13.51	0.781
ALT (IU/L)	28.56 ± 11.52	28.57 ± 10.24	0.997
Diabetes duration (years)	9.47 ± 6.33	8.13 ± 5.57	0.395
Current smoker (%)	14 (31.1)	7 (30.4)	0.954
Current alcohol drinker (%)	27 (62.2)	16 (69.6)	0.549
Medication treatment for, n (%)			
Hypertension	30 (66.7)	17 (73.9)	0.541
Dyslipidemia	34 (75.6)	17 (73.9)	0.882
Benign prostate hyperplasia	20 (44.4)	9 (39.1)	0.675
Number of concomitant antihyperglycemic medication, n (%)			
1	3 (6.7)	3 (13.1)	0.380
2	22 (48.9)	11 (47.8)	0.933
≥ 3	20 (44.4)	9 (39.1)	0.675
Antihyperglycemic drugs			
Sulfonylurea (%)	17 (37.8)	9 (39.1)	0.913
Metformin (%)	40 (88.9)	20 (86.9)	0.814
Dipeptidyl peptidase 4 inhibitors (%)	38 (84.4)	16 (69.6)	0.151
SGLT 2 inhibitors (%)	11 (24.4)	7 (30.4)	0.596
Antihypertensive drugs			
Angiotensin receptor blockers (%)	25 (55.5)	16 (69.9)	0.264
Calcium channel blockers (%)	15 (33.3)	11 (39.1)	0.244
Diuretics (%)	6 (13.3)	5 (21.7)	0.373
Beta-receptor blockers (%)	6 (13.3)	3 (13)	0.973

Data are presented as mean ± standard deviation or proportion (%)

IIEF, International Index of Erectile Function; IPSS, International Prostate Symptom Score; BP, blood pressure; HOMA-IR, homeostatic model assessment-insulin resistance; HDL-C, high-density lipoprotein cholesterol; LDL-C, low-density lipoprotein cholesterol; BUN, blood urea nitrogen; eGFR, estimated glomerular filtration rate; SGLT, sodium-glucose cotransporter

^*^P values were derived using paired Student’s *t*-test or Pearson’s Chi-square test

In the tadalafil group, 3 patients were lost to follow-up and 2 patients discontinued treatment. One patient was lost to follow-up in the placebo group, while another discontinued therapy. Overall, 45 and 23 participants in the tadalafil and placebo groups, respectively, completed the trial (Fig. 1). Baseline characteristics were well-matched between groups at randomization and study completion.

At baseline, the mean HbA1c level in the tadalafil and placebo groups was 6.83 ± 0.77% and 6.77 ± 0.58%, respectively (*P* = 0.747). There were no significant differences between the groups with regard to age, WC, BMI, BP, FBS level, insulin level, C-peptide level, TC level, LDL-C level, eGFR, AST and ALT levels, diabetes duration, smoking status, and alcohol consumption. The groups were comparable with respect to concomitant medications, such as antihyperglycemic and antihypertensive drugs.

### Changes in HbA1c levels

**Fig. 2:**
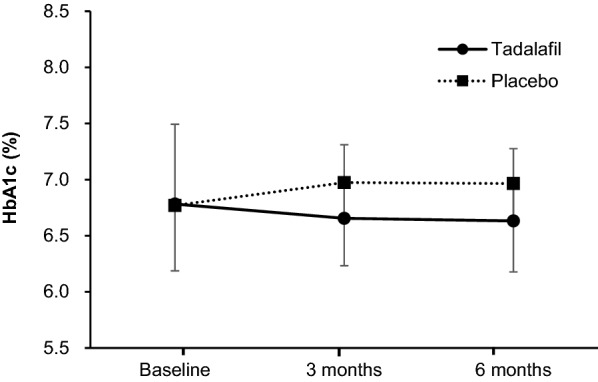
Changes in the HbA1c level over the 6-month study period in the tadalafil and placebo groups. Repeated measures analysis of variance shows significant differences between groups during the 6-month study period (*P* = 0.021)

Repeated-measures ANOVA revealed that the mean HbA1c levels significantly differed between groups over the 6-month study period (*P* = 0.021; Fig. 2). The mean absolute changes in baseline HbA1c levels were − 0.138 ± 0.527% and 0.204 ± 0.492% at 3 months (*P* = 0.012) and − 0.137 ± 0.52% and 0.196 ± 0.691% at 6-months (*P* = 0.030) in the tadalafil and placebo groups, respectively (Table 2).

**Table 2:** Absolute changes in HbA1c in the tadalafil and placebo groups

	3 months from baseline			6 months from baseline		
	Tadalafil (n = 45)	Placebo (n = 23)	*P-*value^*^	Tadalafil (n = 45)	Placebo (n = 23)	*P-*value^*^
Absolute change in HbA1c, %	− 0.138 ± 0.527	0.204 ± 0.492	0.012	− 0.137 ± 0.528	0.196 ± 0.691	0.030

Data are presented as mean ± standard deviation

^*^P values were derived using Student’s *t*-test

### Changes in metabolic parameters

Table 3 shows the changes in metabolic parameters from baseline at 3 and 6 months of treatment in both groups. At 3 and 6 months of treatment, the HOMA-IR score decreased from baseline in the tadalafil group; however, the change was not significantly different from that in the placebo group. In addition, there were no significant differences in WC, BMI, or BP between the groups at 3 and 6 months. At 6 months, the reduction in the FPG level from baseline was significantly greater in the tadalafil group than in the placebo group (− 6.40 ± 28.53 mg/dL vs. 5.35 ± 17.77 mg/dL, *P* = 0.046). At 6 months, the change in the ALT level was − 1.49 ± 12.01 IU/L in the tadalafil group and 6.17 ± 8.39 IU/L in the placebo group (*P* = 0.008). No significant differences were observed in changes in TC, TG, HDL-C, LDL-C, BUN, creatinine, or eGFR between the groups at 6 months.

**Table 3:** Secondary outcomes during the study follow-up period

	3 months from baseline			6 months from baseline		
	Tadalafil (n = 45)	Placebo (n = 23)	*P-*value^*^	Tadalafil (n = 45)	Placebo (n = 23)	*P-*value^*^
IIEF-5	5.96 ± 5.26	0.78 ± 5.82	0.001	6.56 ± 5.32	2.22 ± 5.73	0.003
IPSS	− 4.34 ± 5.91	− 2.77 ± 6.86	0.330	− 4.38 ± 5.86	− 3.08 ± 7.27	0.428
Waist circumference (cm)	0.544 ± 2.45	0.848 ± 1.73	0.598	0.57 ± 3.61	1.44 ± 2.90	0.322
Body mass index (kg/m^2^)	0.10 ± 0.63	1.29 ± 5.81	0.176	0.29 ± 1.62	1.05 ± 5.99	0.429
Systolic BP (mmHg)	2.44 ± 16.67	− 2.78 ± 19.33	0.251	− 2.04 ± 18.42	− 3.65 ± 17.36	0.730
Diastolic BP (mmHg)	1.69 ± 10.88	− 0.35 ± 13.73	0.507	− 2.44 ± 12.58	1.61 ± 13.87	0.229
Fasting plasma glucose (mg/dL)	− 1.40 ± 16.42	4.35 ± 22.09	0.230	− 6.40 ± 28.53	5.35 ± 17.77	0.046
Insulin (IU/mL)	− 0.88 ± 9.14	0.75 ± 8.90	0.484	− 0.78 ± 10.27	0.67 ± 10.92	0.592
C-peptide (ng/mL)	− 0.04 ± 0.88	0.21 ± 0.49	0.218	− 0.05 ± 0.99	0.18 ± 0.60	0.306
HOMA-IR	− 0.46 ± 3.26	0.18 ± 2.62	0.418	− 0.51 ± 3.58	0.32 ± 3.32	0.361
Total cholesterol (mg/dL)				− 6.47 ± 19.80	1.83 ± 1.67	0.106
Triglyceride (mg/dL)				− 12.82 ± 109.27	− 7.13 ± 49.25	0.813
HDL-C (mg/dL)				0.24 ± 6.13	1.0 ± 7.65	0.660
LDL-C (mg/dL)				− 2.67 ± 14.17	3.87 ± 14.54	0.079
BUN (mg/dL)				1.14 ± 5.15	1.79 ± 4.76	0.616
Creatinine (mg/dL)				− 0.047 ± 0.206	− 0.065 ± 0.143	0.701
eGFR (mL/min/1.73 m^2^)				4.34 ± 9.76	5.85 ± 10.86	0.564
AST (IU/L)				0.27 ± 8.52	2.52 ± 15.49	0.440
ALT (IU/L)				− 1.49 ± 12.01	6.17 ± 8.39	0.008

Data are presented as mean ± standard deviation

IIEF, International Index of Erectile Function; IPSS, International Prostate Symptom Score; BP, blood pressure; HOMA-IR, homeostatic model assessment-insulin resistance; HDL-C, high-density lipoprotein cholesterol; LDL-C, low-density lipoprotein cholesterol; BUN, blood urea nitrogen; eGFR, estimated glomerular filtration rate

^*^P values were derived using Student’s *t*-test

### Changes in the International Index of Erectile Function Score and International Prostate Symptom Score

The IIEF-5 and IPSS scores at baseline did not differ between the groups (Table 1). However, improvement in the IIEF-5 score was significantly greater in the tadalafil group than in the placebo group at 3 months (5.96 ± 5.26 vs. 0.78 ± 5.82; *P* = 0.001) and 6 months (6.56 ± 5.32 vs. 2.22 ± 5.73; *P* = 0.003). Moreover, we noted a numerical reduction in the IPSS of the tadalafil group; however, the difference was not statistically significant when compared with the placebo group.

### Adverse events and drug compliance

Tadalafil was well tolerated, and no subject discontinued the study because of treatment-emergent AEs. Overall, a higher percentage of patients reported AEs in the placebo group (*n* = 2, 8.6%) than in the tadalafil group (*n* = 2, 4.4%). The reported AEs were myalgia and senile cataracts in the tadalafil group, while lumbar spinal stenosis, and decreased visual acuity were documented in the placebo group. AEs were mild in severity. The study population showed high medication adherence (> 80%) during the study period.

## Discussion

In this randomized, double-blind, placebo-controlled study, we found that daily administration of 5 mg tadalafil could be associated with improved glycemic control in patients with T2D and ED. Repeated measures ANOVA revealed that the mean HbA1c level significantly differed between the tadalafil and placebo groups during the 6-month study period. Additionally, absolute changes in the baseline HbA1c level at 3 and 6 months of treatment were significantly greater in the tadalafil group than in the placebo group. In addition, the reduction in FPG level from baseline was greater in the tadalafil group at 6 months of treatment than in the placebo group at the same time point.

PDE-5 enzymes are present in various tissues; they are present in penile erectile tissues and blood vessels, platelets, and smooth muscle tissues [26]. Recently accumulated data on chronic usage of low-dose PDE-5 inhibitors indicate that low-dose therapy can provide additional potential benefits in diverse medical conditions, apart from their well-established erectogenic action [27, 28]. However, there are limited placebo-controlled data regarding the effects of PDE-5 inhibitors on glycemic control in patients with T2D. The current study demonstrates that long-term therapy with low-dose tadalafil has a beneficial effect on glycemic control in patients with T2D and ED, as determined using HbA1c and FPG levels.

Tadalafil was first approved for low-dose daily administration at 2.5 or 5 mg for treating ED, and it has a longer duration of action than other PDE-5 inhibitors [29]. Chronic low-dose tadalafil therapy has favorable effects on systemic endothelial dysfunction [30]. Although we did not examine the effect of tadalafil on biomarkers of endothelial dysfunction, tadalafil was found to improve the levels of circulating inflammatory cytokines and chemokines in a diabetic animal model, while improving FPG levels [31]. Moreover, tadalafil improves insulin action on muscle glucose uptake by prolonging NO/cGMP signaling in women with obesity-linked IR [32]. Tadalafil administration was shown to improve β*-*cell function in metabolic syndrome independent of insulin sensitivity [33]. A pilot study has reported improvements in β*-*cell function with tadalafil treatment in individuals with severe obesity [34]. However, the participants in the present study were not severely obese and had good metabolic control, including BMI, WC, and HbA1c values. Therefore, it is difficult to generalize the findings of the present study. Future studies need to evaluate the glycemic effect of daily low-dose tadalafil treatment in obesity.

In the present study, we observed that ALT levels were lower in the tadalafil group at 6 months than in the placebo group. In a previous study, ALT levels were associated with T2D, and glucose-lowering drugs decreased ALT levels as tighter blood glucose levels were achieved [35]. Herein, daily administration of low-dose tadalafil improved erectile function, and there was a statistical difference in IIEF-5 scores between tadalafil and placebo groups. Diabetes-associated ED is predominantly attributed to endothelial dysfunction; however, the precise mechanisms remain poorly understood. Daily therapy with low-dose tadalafil has been approved by the Food and Drug Administration to treat LUTS, which is suggestive of clinical benign prostatic hyperplasia. However, the efficacy of tadalafil for LUTS did not significantly differ from that of the placebo, as reported in other studies [36], which might be due to the small sample size. In the safety assessment, tadalafil was well-tolerated, and no subject discontinued the study due to AEs. The AEs reported were mild in severity.

The current study is the largest double-blinded trial with a longer-term follow-up than previous trials. We observed a small but significant decrease in HbA1c levels after 6 months of therapy. However, this study had several limitations. First, as the present study included a small sample size, there was a limit to obtaining statistically significant results. We also did not adjust for confounding variables, such as lifestyle modifications in diet and physical activity, which may have affected the results. Second, the possible mechanisms that support our results remain unclear. Insulin sensitivity was measured as indexed by HOMA-IR. Still, there is a lack of information on insulin secretion capacity, endothelial markers, and brachial artery flow-mediated dilation. Larger sample, long-term randomized controlled trials are needed to support the hypothesized effects of tadalafil on glycemic control.

## Conclusions

This prospective clinical study found that the daily use of tadalafil 5 mg resulted in more favorable HbA1c and FPG levels than the daily use of placebo after 6 months of treatment. Low-dose tadalafil may be used effectively and safely to improve glycemic control and erectile function in patients with T2D and ED. Nevertheless, future randomized controlled studies with large sample sizes are required to elucidate the underlying mechanisms that could clarify the effects of tadalafil on glycemic control.

## Data Availability

The data used to support the findings of this study are available upon request from the corresponding author.

## Abbreviations

ED: Erectile dysfunction
IIEF: International Index of Erectile Function
NO: Nitric oxide
PDE-5: Phosphodiesterase type 5
cGMP: Cyclic guanosine monophosphate
T2D: Type 2 diabetes
IR: Insulin resistance
OAD: Once a day
BMI: Body mass index
WC: Waist circumference
BP: Blood pressure
FPG: Fasting plasma glucose
TC: Total cholesterol
HDL: High-density lipoprotein
LDL: Low-density lipoprotein
TG: Triglyceride
BUN: Blood urea nitrogen
AST: Aspartate transaminase
ALT: Alanine transaminase
HOMA: Homeostatic model assessment
eGFR: Estimated glomerular filtration rate
IPSS: International Prostate Symptom Score
ANOVA: Analysis of variance
AE: Adverse event
